# A Systematic Review, Meta-Analysis and Meta-Regression on the Effects of Carbohydrates on Sleep

**DOI:** 10.3390/nu13041283

**Published:** 2021-04-14

**Authors:** Angelos Vlahoyiannis, Christoforos D. Giannaki, Giorgos K. Sakkas, George Aphamis, Eleni Andreou

**Affiliations:** 1 Department of Life and Health Sciences, University of Nicosia, 46 Makedonitisas Avenue, Nicosia CY1700, Cyprus; vlahoyiannis.a@unic.ac.cy (A.V.); giannaki.c@unic.ac.cy (C.D.G.); aphamis.g@unic.ac.cy (G.A.); 2 Department of PE and Sport Science, University of Thessaly, 42100 Trikala, Greece; gsakkas@med.uth.gr; 3 School of Sports and Health Sciences, Cardiff Metropolitan University, Llandaff Campus, Western Avenue, Cardiff CF5 2YB, Wales, UK

**Keywords:** glycemic index, glycemic load, nutrition, sleep, polysomnography, actigraphy

## Abstract

This study aimed to assess the effects of quantity, quality and periodization of carbohydrates consumption on sleep. PubMed, SCOPUS and Cochrane Library were searched through October 2020. Data were pooled using random-effects meta-analysis. Eleven articles were included in the meta-analysis which consisted of 27 separate nutrition trials, resulting in 16 comparison data sets (sleep quantity *n* = 11; sleep quality *n* = 5). Compared to high carbohydrate (HCI), low carbohydrate intake (LCI) moderately increased duration and proportion of N3 sleep stage (ES = 0.37; 95% CI = 0.18, 0.56; *p* < 0.001 and ES = 0.51; 95% CI = 0.33, 0.69; *p* < 0.001, respectively). HCI prolonged rapid eye movement (REM) stage duration (ES = −0.38; 95% CI = 0.05, −8.05; *p* < 0.001) and proportion (ES = −0.46; 95% CI = −0.83, −0.01; *p* < 0.001), compared to LCI. The quality of carbohydrate intake did not affect sleep stages. Meta-regression showed that the effectiveness of carbohydrate quantity and quality in sleep onset latency was significantly explained by alterations of carbohydrate intake as a percentage of daily energy intake (R^2^ = 25.87, *p* = 0.018) and alterations in the glycemic load (R^2^ = 50.8, *p* = 0.048), respectively. Alterations in glycemic load partially explained the variance of the effectiveness of carbohydrate quality in sleep efficiency (R^2^ = 89.2, *p* < 0.001) and wake after sleep onset (R^2^ = 64.9, *p* = 0.018). Carbohydrate quantity was shown to affect sleep architecture, and especially N3 and REM sleep stages. Alterations in both quantity and quality of carbohydrate intake showed a significant effect on sleep initiation. Variations in carbohydrate quality significantly affected measures of sleep continuation. Further studies are needed to assess the effect of long-term carbohydrate interventions on sleep.

## 1. Introduction

Sleep constitutes a lifelong element of human existence. It is defined as a reversible state of decreased or absent consciousness that initiates from wakefulness and evolves to light, deep and rapid eye movement (REM) sleep stages [1]. Alterations in sleep-related parameters are often part of physiological responses that may be induced by nutrition interventions and might be translated into modifications in sleep architecture [2], quantity or continuation [3].

The required sleep quantity varies among individuals and its adequacy is reflected by the absence of sleep-induced or sleep-related health issues, daytime dysfunction or sleepiness [4]. Insufficient sleep traits have been increased over the last years [5,6]. Its association with numerous diseases [7] underlined the necessity to examine practical and effective approaches, including manipulation of nutritional aspects to optimize sleep [8]. Various nutrition interventions have been studied for their effects on sleep-related parameters. Interestingly, both dietary supplements (e.g., tryptophan [9]) and consumption of whole foods (e.g., kiwifruit [10], tart cherry juice [11]) have shown promising effects on improving sleep quality.

Animal models revealed that hormones [12,13] and peptides [14] entrain the body’s circadian system and affect sleep. Since the circadian system facilitates most human behavioral and physiological processes, observational studies in humans have tried to investigate the effect of macronutrient intake on sleep quality and stated that alterations in the distribution and periodization of macronutrients are associated with sleep optimization [15]. Towards this direction, the findings of nutrient–sleep interactions show to be positive but are yet unverified, with unsettled biological mechanism explanations [16].

The acute manipulation of dietary carbohydrate (CHO) has been highlighted over the years with regards to its effect on sleep-related parameters. Carbohydrates are considered to be a critical macronutrient for sleep, not only because they serve as a primary source of energy for all human cells, but due to their relationship with the brain function and sleep-related hormonal regulation [17]. Glucose metabolism is highly interrelated with sleep [18] by modifying the plasma tryptophan concentration [19], a precursor of serotonin and melatonin, which, in turn, has a significant effect on sleep initiation and continuation [20]. Two recent reviews suggest that dietary melatonin intake (either from fruits and vegetables [21], or milk and cherry juice [22]) could have sleep-promoting effects. Research shows that carbohydrates are associated with alterations in sleep onset latency [23], sleep time [3], sleep continuity [3] and sleep stages [24]. Nonetheless, the effect of CHO intake in sleep has not been systematically reviewed yet. Furthermore, since dietary CHO intake from individual studies may vary in quantity, quality or timing/duration of intervention, it is of utmost importance for these factors to be taken into account and analyzed distinctively. Therefore, this review aimed to assess the effects of CHO consumption in sleep through systematic review and meta-analysis. Specifically, the objectives of this review were to:

- Examine the effect of the quantity of CHO consumption on sleep quantity, continuity and architecture.
- Address potential effects of the quality of CHO (e.g., Glycemic Index or Glycemic Load) on sleep quantity, continuity and architecture.
- Investigate the effect of the timing of CHO consumption on sleep quantity, continuity and architecture.

## 2. Methodology

### 2.1. Information Sources and Search Strategy

The review was conducted according to the PRISMA guidelines [25] and the research strategy performed using the PICO model (Population: healthy participants with normal weight, aged above 18 years old; Intervention/Comparison: groups with alterations in carbohydrate consumption; Outcome: Nocturnal sleep-related parameters obtained from polysomnography (PSG), sleep electroencephalography (EEG) or actigraphy). Three electronic databases (PubMed, SCOPUS and Cochrane Library) were systematically searched by two of the authors (A.V. and E.A.) for studies published from inception to 10 October, 2020. For all databases, search terms included the following combination of keywords: “(((((((carbohydrate*) OR CHO) OR glycemic index) OR glyceamic index) OR glycemic load) OR glyceamic load)) AND ((((((((((sleep) OR sleep patterns) OR sleep characteristics) OR sleep architecture) OR sleep habits) OR sleep quality) OR sleep quantity) OR sleep duration) OR sleep efficiency) OR sleep stage).” Retrieved records from Pubmed search were limited according to species (Humans) and language (English). Accordingly, retrieved records from the SCOPUS search were limited according to source type (Journal), document type (Article) and language (English). Retrieved records from the Cochrane Library were limited to trials. A supplementary search for relevant studies was conducted from the reference list of the screened manuscripts.

### 2.2. Eligibility Criteria

The eligibility of retrieved records was screened according to the following criteria: (1) studies were written in English, (2) carbohydrate intervention was manipulated by the researchers and described adequately, (3) nocturnal sleep-related parameters were measured objectively via polysomnography (PSG), electroencephalography (EEG), actigraphy or a combination of these methods and (4) participants aged above 18 years old. Only clinical trials were considered for inclusion. Articles were excluded if: (1) participants had a history of major diseases, (2) nutrition intervention was affected by other intervention or condition, and (3) sleep-related data were assessed after travel, other manipulated intervention (e.g., altitude or light exposure) or any other dietary or pharmacological intervention.

### 2.3. Study Selection and Data Extraction

**Figure 1:**
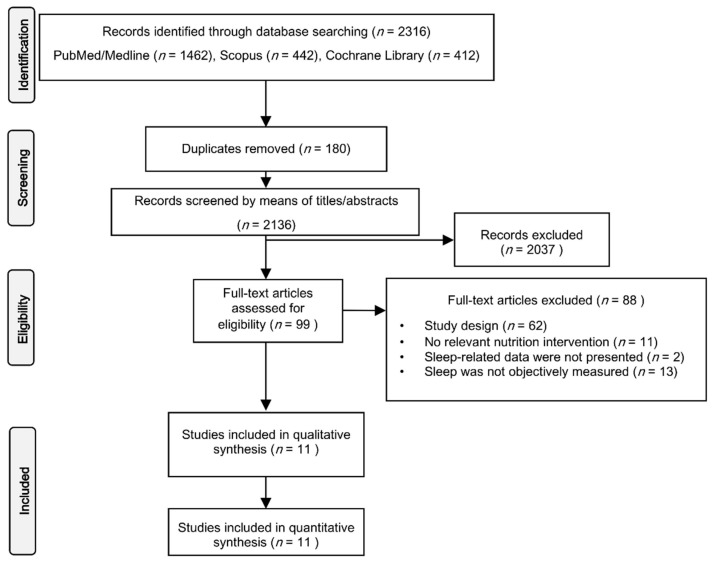
PRISMA flow chart for study selection.

With the inclusion criteria, the identified articles were first screened by their titles and abstracts, followed by a full-text screening. After the removal of duplicated records from the retrieved studies of the initial search, titles abstracts and full manuscripts were assessed with a hierarchical approach. The study selection flowchart is illustrated in Figure 1. The database search and study selection were performed by two reviewers (A.V. and E.A.) independently. Any discrepancies in the selection process were discussed until a consensus was reached.

A standardized data extraction form was employed to obtain the following: (1) publication details: name of first author and year of publication, (2) study design and participants’ characteristics (sample size and sex), (3) nutrition intervention data: (i) CHO intervention classification (quantity, quality and timing) and (ii) macronutrient analysis of the dietary intervention, (4) sleep-related parameters (further described below).

### 2.4. Evidence Quality Appraisal

All studies were assessed for their methodological quality using the QualSyst tool (Supplementary Table S1) [26]. Two reviewers (A.V. and E.A.) used this 14-item checklist to assess the included studies. When a criterion was satisfied, a score of 2 (=yes) or 1 (=partial) was awarded. Otherwise, a score of 0 (=0) was awarded the corresponding item. The overall score was calculated as the total score, divided by the total possible score. Study evidence quality ratings were classified as weak (≤55%), moderate (55–75%) or strong (≥75%).

### 2.5. Definitions of Nutrition-Related and Sleep-Related Parameters

Nutrition interventions were categorized based on the variance of the quantity of CHO consumption, the quality of CHO consumption or the timing of CHO consumption during a short-term (one or more days) or an acute (less than two meals) intervention. The quantity of CHO was defined by the amount (g) of CHO intake, and lower CHO intakes (LCI) were compared to higher CHO intakes (HCI). The quality of CHO consumption was based on meals’ Glycemic Index (GI) or Glycemic Load (GL). The GI is a ranking of carbohydrates on a scale from 0 to 100 according to the extent to which they raise blood sugar (glucose) levels after eating [27]. Foods with GI ≥ 70 are classified as high, foods with GI 56–69 as moderate and foods with ≤55 as low GI. Glycemic load is a combination of the quantity and quality of carbohydrates. It is calculated as: GL = GI × Carbohydrate (g) content per portion ÷ 100. Similar to the GI, the GL of a food can be classified as low (≤10), medium (11–19) or high (≥20). In the case of acute CHO intervention, the timing was defined as the timing of meal consumption related to the usual bedtime.

The selected nutrition parameters were selected in order to systematically evaluate their effect on the following sleep variables:

- Total Sleep Time (TST): The total time spent asleep during the recording night.
- Sleep Efficiency % (SE): The percentage of the Ratio of Total Sleep Time (TST) to Time in Bed.
- Sleep Onset Latency (SOL): Time from lights out until sleep onset (generally defined as first epoch of sleep Stage 2).
- Wake After Sleep Onset (WASO): The duration of wake during the night after initial sleep onset. This term was used in parallel with “Total Wake Time” because of their similarity and the interchangeable use of them in the shortlisted studies.
- REM Onset Latency (ROL): the interval between sleep onset and the onset of the first REM sleep period.
- Stage 1 (N1): Duration of sleep Stage 1, presented as minutes or percentage of TST.
- Stage 2 (N2): Duration of sleep Stage 2, presented as minutes or percentage of TST.
- Stage 3 (N3): Duration of Slow Wave Sleep (SWS), presented as minutes or percentage of TST.
- REM Sleep (REM): Duration of REM generally presented as minutes or percentage of TST.

### 2.6. Data Synthesis

Data were extracted from as many sets as possible, separating data sets for each type of intervention. Extracted data sets were categorized, analyzed and interpreted according to CHO intervention quantity, quality and duration/timing of CHO intervention. The mean and standard deviations (SD) for all data were recorded. Where required, SD was calculated from reported Confidence Intervals (CIs) or imputed as the average SD of similar studies, as proposed by the Cochrane Handbook for Systematic Reviews of Interventions [28]. When data were reported in different units, they were transformed accordingly for purposes of uniformity. In the case of merged N1 and N2 stages of sleep (as light sleep), the results are presented but not included in the calculation of general or subgroup mean. In case of sleep scoring with five stages (REM, N1, N2, N3 and N4), the N3 and N4 stages were combined [1].

For all analyses, the comparator group received CHO with different quantity, quality or timing of CHO consumption, or ingested a similar supplement or was placebo controlled. If a study included two CHO intervention groups and a placebo-control group that were all part of the nutrition intervention, all the intervention groups were retrieved and compared pairwise. Where the change in SD (ΔSD) was not reported, it was imputed from before and after intervention SD. The correlation coefficient (corr) was calculated according to the Cochrane Handbook for Systematic Reviews of Interventions [28]:

Corr = (SD_pre_^2^ + SD_post_^2^ − SD_change_^2^)/(2 × SD_pre_ × SD_post_)

In addition to the effect of CHO on sleep-related parameters, two main domains (discriminated accordingly by the reporting of sleep stages in minutes or % of TST) were created:

1. Sleep Depth (min): defined by shorter duration of N1 and N2 sleep stage, and longer duration of N3 sleep stage in minutes.
2. Sleep Depth (%): defined by lower TST percentage of N1 and N2 sleep stage, and higher percentage of N3 sleep stage.
3. REM attainment (min): defined by shorter ROL and increased REM duration in minutes.
4. REM attainment (%): defined by shorter ROL and increased % REM.

### 2.7. Statistical Analyses and Meta-Analytic Calculations

Hedges g’ effect size (ES) with 95% CIs was calculated to reflect standardized differences in means for individual sleep-related parameters between two CHO interventions or before and after CHO intervention. Scores were rated as small (ES < 0.4), moderate (ES = 0.4–0.7) or large (ES > 0.7). For interpretation purposes, effect sizes were further transformed in the corresponding units using pooled SD for each CHO domain (quantity or quality). For the sleep domains, single variable ES with SEs was inserted into one meta-analysis for each sleep domain, after adjustment for direction (For Sleep Depth: N1 and N2 ES were multiplied by −1 and for REM attainment: ROL ES was multiplied by −1). Thus, each study could contribute multiple times to the same domain, according to the number of variables the particular study reported in the domain. Only studies that reported all sleep variables for each sleep domain were included in this specific analysis.

Multiple sensitivity analyses such as Q and I^2^ statistics were performed to determine if any of the results were influenced by heterogeneity. An I^2^ value > 50% was regarded as evidence of substantial heterogeneity and, thus, a random-effect model was then preferred to a fixed-effect model. Furthermore, since heterogeneity statistic I^2^ was proposed that can be biased in small meta-analyses [29], funnel plots were assessed graphically for publication bias (supplemental material; Figures S1 and S2) calculated by Meta-Essentials: Workbook for meta-analysis [30]. Meta-analyses were conducted when at least two data sets were available. In an effort to further explore potential sources of heterogeneity, meta-regressions were performed for all the primary outcomes using a random-effects model. For studies investigating the effect of carbohydrate quantity on sleep, the differences of the percentage of carbohydrate dietary intake between trials was set as moderator. For studies investigating the effect of carbohydrate quality on sleep, the differences of glycemic load of the pre-bed meal were set as moderator. A minimum of four data sets were required to perform the regression analysis. Meta-regression was chosen instead of subgroup analysis in order to further explore potential collinearity between the effectiveness of each intervention and the continuous covariate (moderator). A significance level of *p* < 0.05 was set throughout the meta-analysis.

## 3. Results

### 3.1. Studies’ Selection

The literature search resulted in 2316 studies (Figure 1). After duplicate removal, 2136 studies were initially screened by title and abstract. Ninety-nine studies were short-listed and assessed for eligibility. Through full-text screening, 88 articles were excluded, resulting in 11 articles identified that were included in the qualitative synthesis and quantitative analysis.

### 3.2. Studies’ Characteristics

The characteristics of the 11 studies are presented in Table 1. Included studies were published from 1975 to 2019. Eight studies used a cross-over design [3,23,24,31,32,33,34,35] and three used a single group pre-post design [2,36,37]. Three studies were conducted in the United Kingdom [24,33,36], three in Australia [2,23,34], three in United States [31,32,37], one in Cyprus [3] and one in Brazil [35]. According to the “QualSyst” scale, evidence quality of five studies [2,24,35,36,37] were classified as moderate and six studies [3,23,31,32,33,34] were classified as strong. None of the studies was scored as having weak evidence quality. Analytical scoring data are presented in Supplemental Table S1.

The included sample was 179 participants (males *n* = 59; females *n* = 6; combined -without clear sex segregation *n* = 114), with ages ranging from 8 to 45 years and normal BMI. Overall, participants were tested in 27 trials of nutrition intervention, resulting in 16 comparison data sets (Table 2). Specifically, eleven comparisons investigated the effect of CHO quantity on sleep [2,24,31,32,33,36,37] and five investigated the effect of CHO quality on sleep [3,23,34,35]. Of these data sets, seven studied an acute CHO intervention [3,23,33,34,35], eight studied a short-term (over a period of few days) CHO intervention [2,24,31,32,36,37] and two data sets compared an acute response to a few days of CHO intervention [2]. In Table 2, the consumption of the other two macronutrients, fats and proteins is also presented, as well as the dietary energy content. It is noted that, in the majority of the studies, the energy content for both HCI and LCI trials was similar. In this manner, between trials dietary fats were substituted by carbohydrates, with no significant modifications in protein intake. Sleep was monitored either with polysomnography or EEG recordings, except for three studies [31,32,35] that measured sleep with actigraphy. The extracted sleep variables for each study are presented in the Table 3.

### 3.3. Effect of Carbohydrate Quantity on Sleep

**Figure 2:**
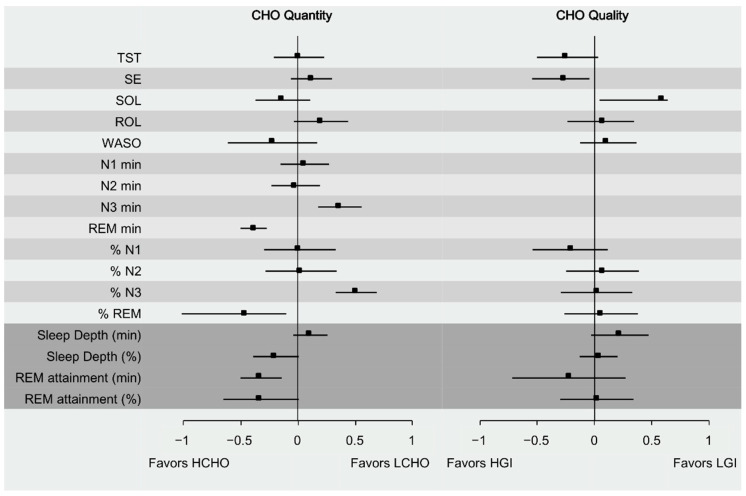
Summary representation of the combined effect sizes with 95% Carbohydrate Intake (CI) for all sleep domains.

Meta-analytic calculations for all sleep variables are presented in Table 4 and Figure 2. Total Sleep Time, SE, SOL, ROL, N1 (min and %) and N2 (min and %) and Sleep Depth (min) did not differentiate significantly between HCI or LCI. Compared with HCI, LCI intake moderately increased N3 stage both in duration (minutes) and proportion (ES = 0.37; 95% CI = 0.18, 0.56; I^2^ = 0%; *p* < 0.001 and ES = 0.51; 95% CI = 0.33, 0.69; I^2^ = 0%; *p* < 0.001, respectively). HCI intake moderately prolonged REM stage duration (ES = −0.38; 95% CI = 0.05, −8.05; I^2^ = 0%; *p* < 0.001) and its proportion (ES = −0.46; 95% CI = −0.83, −0.01; I^2^ = 0%; *p* < 0.001), compared to LCI intake. REM attainment (both min and %) increased in HCI compared to LCI trial (ES = −0.31; 95% CI = −0.47, −0.15; I^2^ = 0%; *p* < 0.001 and ES = −0.32; 95% CI = −0.57, −0.08; I^2^ = 21.5%; *p* = 0.003, respectively), as well as Sleep Depth % (ES = −0.19; 95% CI = −0.57, −0.08; I^2^ = 0%; *p* < 0.001), with a small effect size.

Large heterogeneity was observed for SOL. Funnel plot suggested that the effects of CHO quantity on SOL may be affected by potential publication bias. Sensitivity analysis was performed, excluding one study [37] that had the largest effect size and could be potentially an outlier. By excluding this data set, heterogeneity was reduced to 0% without altering the results between the two conditions.

### 3.4. Effect of Carbohydrate Quality on Sleep

Carbohydrate quality did not significantly affect TST, SE, SOL, ROL, WASO, N1 (%), N2 (%) REM (%), Sleep Depth (%) or REM attainment (both min and %). Data were not adequate to perform metanalytic calculations for all sleep stages that were reported in minutes. LGI pre-bed meals increased Sleep Depth (min) with a small effect size (ES = 0.22; 95% CI = −0.03, 0.47; I^2^ = 0%; *p* = 0.022 and ES = 0.22; 95% CI = −0.03, 0.47; I^2^ = 0%; *p* = 0.022, respectively).

Because of the large heterogeneity in SE, SOL and WASO, funnel plots were inspected graphically to evaluate potential publication bias. It was shown that except for SOL, there was no evidence of publication bias. Sensitivity analysis with exclusion of the data set with the larger effect size for SE, SOL, WASO, as a potential outlier, showed that results and I^2^ did not substantially change.

### 3.5. Carbohydrate Timing and Sleep

According to carbohydrate timing, no metanalytic calculations could be performed due to the inadequate data sets. Specifically, from the seven data sets of comparisons of CHO quantity, two sets of one study [33] were classified as an acute nutrition intervention and seven as short-term [2,24,31,32,36,37], with a maximum of four days of the adoption of a nutrition protocol [31,32,38]. From the five data sets of comparisons between CHO quality, all data studied acute nutrition interventions on sleep [3,23,34,35]. Thus, the only comparison that could be applied is between the short-term study of Porter and colleagues, and the rest were trials that studied CHO quantity in sleep. All the included studies show that both a pre-bed meal LCI and a LCI diet over a period of a maximum of four days may decrease REM and increase N3 sleep stages compared to a HCI meal or diet, respectively. According to the acute effect of the pre-bed meal, Afaghi and colleagues showed that 4 h pre-bed HGI meal could decrease SOL more than a similar meal consumed 1 h before bedtime [23].

### 3.6. Meta-Regression Analyses

The large heterogeneity observed for SOL in the studies that investigated alterations in carbohydrate quantity consumption was further explored with meta-regression analysis. It was found that % energy alterations in CHO intake explained a part of the variance in the effectiveness of CHO quantity in SOL (coefficient = 0.007, SE = 0.003, 95% CI = 0.0003, 0.014, z = 2.365, *p* = 0.018, Adj. R^2^ = 25.87). No other variance, with regards to sleep continuation or architecture, was significantly explained by the alteration of the % energy from CHO intake between trials.

In an effort to understand the increased heterogeneity for SE, SOL and WASO in the studies that investigated the effects of carbohydrate quality in sleep, meta-regressions were performed according to the alterations of the glycemic load between trials. These analyses significantly explained part of the variance of the effectiveness of CHO quality in SE (coefficient = −0.018, SE = 0.003, 95% CI = −0.028, 0.009, z = −5.490, *p* < 0.001, Adj. R^2^ = 89.2), SOL (coefficient = 0.028, SE = 0.014, 95% CI= −0.011, 0.067, z = 1.971, *p* = 0.048, Adj. R^2^ = 50.8) and WASO (coefficient = 0.014, SE = 0.006, 95% CI= −0.002, 0.030, z = 2.35, *p* = 0.018, Adj. R^2^ = 64.9). No other variance with regards to sleep continuation of architecture was significantly explained by the alteration of the glycemic load between trials.

It was not possible to perform a similar meta-regression analysis on the remaining set of sleep-related data and carbohydrate periodization due to the lack of available data.

## 4. Discussion

Over the last decades, there has been a great interest in the relationship of diet on sleep. The current meta-analysis of clinical trials showed that CHO intake could significantly affect both sleep architecture, sleep initiation and continuation. In particular, a lower quantity of CHO intake significantly lengthens N3 stage sleep proportion and duration compared to higher CHO consumption. Increased dietary CHO intake significantly prolonged REM stage sleep compared to lower CHO intake. Small effects were also observed for increased CHO quantity and REM attainment and sleep depth compared to lower CHO intake. The quality of CHO intake did not show any significant effect on sleep stages. Sleep onset latency showed to be affected by both carbohydrate quantity and quality. Alterations in the quality of carbohydrate intake showed a significant effect on measures of sleep continuation.

**Figure 3:**
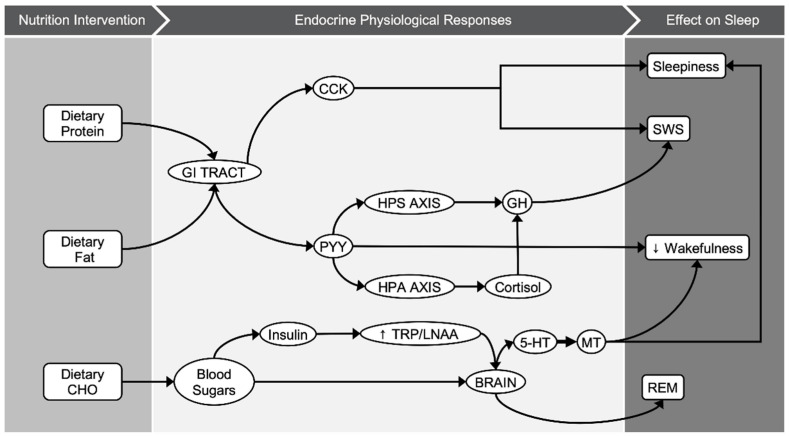
Graphical illustration of potential biological pathways behind macronutrients–sleep interactions.

Diet-induced alternations in CHO quality and quantity have shown some promising results with regards to sleep-related parameters, based on the limited data. Replacing a typical diet, one that is mainly composed of CHO as main source of energy intake, with a LCI diet showed increases in both duration and proportion of deep sleep [2,24,33,36,37]. Evidence from genetic studies have shown that sleep duration is associated with rs2031573 and rs1037079 alleles, with relevance to shorter sleep [39]; however, it is not established yet whether diet modifications could affect the epigenetics of those genes and consequently the quality of sleep. The effect of CHO intake in deep sleep could be attributable to various biological mechanisms related to diet-dependent hormonal regulation (Figure 3). It is proposed that dietary fat and protein (or their digestion products) stimulate cholecystokinin (CCK) release to a greater extent than CHO [40,41]. CCK is produced in a number of tissues in humans, including enteroendocrine cells of duodenum, and is distributed in the brain as well [42]. In healthy male and female volunteers, a LCHO meal led to higher postprandial CCK concentrations and increased subjective feelings of sleepiness [40]. Nonetheless, the relationship between CCK and sleep was first demonstrated in animal experiments [12,13]. In both rats and rabbits, intraperitoneal injection of cholecystokinin showed a dose–response relationship between circulating CCK levels and N3 sleep stage (referred as SWS or “deep sleep”). Following the same line, both dietary fat and CCK may result in higher levels of peptide-tyrosine-tyrosine (PYY) [41]. PYY is a peptide released in the gastrointestinal tract and it is usually studied for its role in appetite regulation, and especially its anorexigenic effect [41]. In animal models, nocturnal intraperitoneal administration of PYY decreased wakefulness and enhanced NREM sleep [14]. In contrast to PYY, ghrelin is an appetite-stimulating hormone that is linked to sleep-wake behavior [43]. The magnitude of the effect of ghrelin on sleep is differentiated according to sex, being greater in males than females [44]. It is proposed that ghrelin stimulates the activity of the hypothalamic-pituitary-adrenal and hypothalamic-somatotrophic axes, altering growth hormone (GH) and cortisol levels [43], both hormones that are involved in sleep regulation. N3 sleep stage coincides with approximately 70% of GH pulses, and the amount of N3 sleep stage is positively related to the amount of GH secretion during these pulses [45]. Moreover, cortisol administration in both young [46] and elder adults [47] is positively linked with endogenous GH secretion and N3 sleep stage. In this meta-analysis, all individual studies that modified CHO quantity acutely or in the short term showed increases in SWS [2,24,33,36]. The pooled results suggest that a LCHO pre-bed meal ranging between 0–47 g of CHO or a LCHO diet ranging between 2–100 g of CHO increased N3 sleep stage by 8.5 min or by 3.2% compared to a HCHO pre-bed meal ranging between 130–196 g of CHO or a HCHO diet ranging from 240–600 g of CHO. As N3 occupies approximately 20% of TST [1], an increase of 3.2% is translated to 16% of the time that is spent in this specific sleep stage.

On the other hand, this meta-analysis revealed that higher CHO intake increased REM sleep compared to lower CHO consumption. Frequently, the proposed model behind this effect is attributed to the insulin effect on tryptophan regulation. It is known that both the quantity [48] and the quality [19] of dietary CHO affect tryptophan (TRP) availability and the ratio of TRP to large neutral amino acids (LNAA) in plasma. Postprandial insulin secretion triggers peripheral uptake of LNAA and inhibits the release of peripheral amino acids. However, TRP is largely bounded to albumin and as a consequence, the concentration of the other competing LNAAs is reduced and TRP level in plasma increases [48,49]. As TRP/LNAA ratio and TRP availability increase, the traverse of TRP through the blood-brain barrier is favored. In the brain, increased TRT levels induce synthesis of serotonin, a precursor of melatonin. Free plasma-tryptophan correlates positively with the amount of REM sleep among healthy individuals [50]. Parker and Rossman in 1971 showed that glucose infusion during the first 3 h of sleep increased REM and decreased light sleep [51]. It could by hypothesized that in a specific time frame, increases in REM sleep result in a reciprocal reduction of NREM sleep. Furthermore, a possible explanation could be that given that the metabolic and glucose demands are higher during REM sleep compared to SWS [52,53], REM sleep is reduced when dietary CHO is restricted and vice versa. This positive relationship of CHO amount and REM sleep stage was consistent for the included studies, suggesting that increased CHO intake either in a pre-bed meal (ranging from 130 g to 196 g) or in the daily dietary plan (ranging from 240 g to 600 g) prolonged REM sleep by 8.9 min or 2.6%. Taking into account the norm of 25% of TST spent in the REM stage, the increase of 5.6% reflects a proportion of 22.4% of total REM sleep.

According to CHO quality, it has often been suggested that a HGI may be beneficial for improving sleep-related parameters. The included studies showed ambiguous results according to CHO quality and sleep. All experiments manipulated the GI of the last or two meals before bedtime. While some of the studies found significant improvements of HGI in SOL, SE or WASO [3,23], others found weak or no effects [34,35]. The studies that found that sleep-related parameters were optimized after a HGI pre-bed meal attributed these effects on potentially increased melatonin synthesis. In agreement, a meta-analysis showed that exogenous melatonin administration induced similar sleep-related alterations to a pre-bed HGI meal, such as a reduction in SOL, increases TST and SE [20]. A recent review suggested that food sources of melatonin such as milk and tart cherries could act as a safeguard for sleep-related issues by improving sleep quality [22]. Nevertheless, none of the included studies in the current meta-analysis found any significant effect on sleep architecture. Pooled effect sizes showed a small effect on sleep depth in favor of LGI diets/meals that may be attributed to small decreases of the proportion of N1 and N2 sleep rather than increases in the N3 sleep stage. Because of the limited information, we were unable to conduct a meta-analysis for sleep stages or domains of sleep depth or REM pressure, as only two studies reported the related data [3,34].

The apparent lack of causality for CHO quantity and quality with other sleep variables such as sleep onset latency, sleep duration or continuation led to the hypothesis that the aforementioned studies included good sleepers and thus, the potential for sleep improvement was limited. Nevertheless, meta-regression analyses showed that the degree of differentiation of carbohydrate quantity showed a linear association with sleep onset latency and that alteration in GL explained a part of the variance of several sleep continuation parameters. Towards this direction, a previous investigation of the effect of CHO quality as a post-exercise meal in good sleepers showed several improvements in both sleep quality and quantity [3]. This optimization of sleep efficiency and continuity after a HGI meal intervention as a post-workout meal could be attributed to the increased post-exercise insulin sensitivity [54], which may promote greater effects on plasma TRP availability. Thus, potential covariates are of further interest and should be treated with caution when investigating the effect of nutrition interventions in sleep.

Results of the present meta-analysis support the employ of CHO quantity as a reasonable non-pharmacological tool for modification of sleep architecture, if needed. As appropriate, high CHO quantity could be used to potentially increase REM, while lower CHO could be useful to increase N3 sleep stage. Since 1980, SWS has been suggested to be associated with widespread bodily restorative functions, while REM sleep may be more associated with synthetic processes of brain reorganization and repair [55]. SWS is known to be a marker of sleep homeostasis [56], and its nocturnal appearance, with the longest episode usually in the first sleep cycle, is tied to growth hormone release [57], implying its restorative properties. In healthy adult volunteers, selective SWS deprivation led to decreases in behavioral performance and a significant rebound of SWS on the recovery night [58]. Contrarily, REM stage sleep deprivation seems not to be detrimental to behavior and psychomotor function in healthy subjects or those who are schizophrenic or depressed [59]. However, disturbed melatonin metabolism in combination with selective lesions of acetylcholine neurons in the pedunculopontine tegmental nucleus that modulate both REM and arousal may be involved in sleep disorders in individuals with Cockayne syndrome [60].

Moreover, the practical application of this intervention may extend towards to the optimization of sleep quality in poor sleepers via the modification of the prescribed CHO quality. Scientific evidence indicated that three main indicators of poor sleeping (SOL, SE and WASO) were significantly improved in response to CHO quality manipulation. Hence, it could be hypothesized that both sleep initiation and sleep continuation could be also optimized in poor sleepers via other means. Since the prevalence of poor sleeping traits increases dramatically [5], these results are of further importance, and relevant studies should be conducted. Several potential limitations of this meta-analysis need to be considered. A common constraint in meta-analyses is heterogeneity of the included studies [61]. In the current meta-analysis, the estimates for clinical trials that monitored sleep after a CHO-related intervention were relatively homogenous, and efforts have been made to compare clinical trials with similar design. Extracted results did not affect potential outliers and any sensitivity analysis did not contribute to the formation of altered results. Furthermore, nutrition timing seems to affect sleep potentially, but to date only one eligible trial was identified that compared the timing of dietary CHO in sleep [23]. Thus, the lack of studies investigating CHO timing do not allow for drawing safe conclusions of these results. Furthermore, the included studies did not report an adequate number of data sets differentiating according to age or sex. Thus, more studies are needed to identify the mediating role of age between the link of carbohydrates and sleep during the lifespan, as well as the impact of BMI on sleep and nutrition interactions. Additionally, more evidence from controlled clinical trials on the effects of long-term sleep optimizing interventions are desirable. In addition, with regard to CHO quality, only acute-effect interventions were studied, and it was further observed that no long-term effects of CHO quantity and quality in sleep had been studied yet.

## 5. Conclusions

The current results highlight the effect of nutrition, and especially carbohydrates, on sleep. It was observed that a lower quantity of CHO intake significantly increases N3 stage sleep and higher dietary CHO intake significantly prolongs REM stage sleep. The quality of CHO intake did not show any significant effect on sleep stages. The effectiveness of carbohydrate quantity and quality in sleep onset latency was significantly explained by alterations of carbohydrate intake as a percentage of daily energy intake and alterations in the glycemic load, respectively. Changes in glycemic load partially explained the variance of the effectiveness of sleep quality in sleep efficiency and wake after sleep onset. To date, there is no clear interpretation of the relevant biological mechanisms. Results for CHO quantity and sleep stages are promising and need to be further addressed in future studies with long-term interventions in different age groups for both genders.

## Figures and Tables

**Table 1:** Characteristics of included studies.

Author, Year, Reference	Country	Study Design	N	Sex	Age (Year)	BMI (kg/m^2^)	Quality Score
Phillips et al., 1975 [24]	UK	Crossover	8	M	NR	18.5–25	Moderate
Porter and Horne, 1981 [33]	UK	Crossover	6	M	NR	NR *	Strong
Kwan et al., 1986 [36]	UK	Single Group pre-post design	6	F	20–23	19–24	Moderate
Afaghi et al., 2007 [23]	Australia	Crossover	12	M	18–35	18.5–25	Strong
Afaghi et al., 2008 [2]	Australia	Single Group pre-post design	14	M	18–35	23.4 ± 1.9	Moderate
Jalilolghadr et al., 2011 [34]	Australia	Crossover	8	M & F	8–12	18.9 ± 2.2	Strong
Lindseth et al., 2013 [31]	USA	Crossover	44	NR	19–22	24.8 ± 3.5	Strong
Lindseth and Murray, 2016 [32]	USA	Crossover	36	M & F	20.9 ± 1.9	24.6 ± 4.1	Strong
St-Onge et al., 2016 [37]	USA	Single-Group pre-post design	26	M & F	30–45	22–26	Moderate
Vlahoyiannis et al., 2018 [3]	Cyprus	Crossover	10	M	18–26	24.9 ± 4	Strong
Daniel et al., 2019 [35]	Brazil	Crossover	9	M	18.0 ± 0.7	23.9 ± 1.5	Moderate

* Study reported that subjects were within ±5 kg of ideal body weight; M: Males; F: Females; NR: Not Reported.

**Table 2:** Nutrition Intervention Data.

Author, Year, Reference	Type of Intervention (A = CHO Quantity; B = CHO Quality; C = CHO Timing)	Duration of Intervention (A = Acute; B = Short -Term)	Timing of Intervention (A = 45 − 1 h; B = 4 h)	Trial	Nutrition Intervention Macronutrient Analysis						
					Kcal	CHO (g)	CHO (%)	Fat (g)	Fat (%)	Protein (g)	Protein (%)
Phillips et al., 1975 [24]	A	B	NA	HCI	2997	600.0	80.1	33.0	9.9	75.0	10.0
		B	NA	LCI	2995	100.0	13.4	255.0	76.6	75.0	10.0
Porter and Horne, 1981 [33]	A	A	A	HCI	714	130.0	72.8	18.0	22.7	8.0	4.5
		A	A	ZCI	0	0.0	0.0	0.0	0.0	0.0	0.0
		A	A	LCI	401	47.0	46.9	21.0	47.1	6.0	6.0
Kwan et al., 1986 [36]	A	B	NA	HCI	1929	240.0	49.8	83.0	38.7	64.0	13.3
		B	NA	LCI	2066	49.0	9.5	164.0	71.4	103.0	19.9
Afaghi et al., 2007 [23]	B & C	A	A	HGI (1 h)	764	173.0	90.6	1.3	1.5	15.0	7.9
		A	B	HGI (4 h)	764	173.0	90.6	1.3	1.5	15.0	7.9
		A	B	LGI (4 h)	764	173.0	90.6	1.3	1.5	15.0	7.9
Afaghi et al., 2008 [2]	A	A & B	B	HCI	1090	196.0	71.9	15.0	12.4	42.0	15.4
		A	B	LCI (acute)	1090	2.0	0.7	74.0	61.1	103.0	37.8
		A & B	B	LCI (2 days)	1090	2.0	0.7	74.0	61.1	103.0	37.8
Lindseth et al., 2013 [31]	A	B	NA	HCI	NR	NR	56.0	NR	22.0	NR	22.0
		B	NA	HCI	NR	NR	50.0	NR	35.0	NR	15.0
		B	NA	LCI	NR	NR	22.0	NR	56.0	NR	22.0
Jalilolghadr et al., 2011 [34]	B	A	A	HGI	238.4	45.1	75.6	0.6	2.3	13.2	22.1
		A	A	LGI	277	25.9	37.3	13.6	44.2	12.8	18.5
Lindseth and Murray, 2016 [32]	B	B	NA	HCI	NR	NR	80.0	NR	10.0	NR	10.0
		B	NA	HCI	NR	NR	50.0	NR	35.0	NR	15.0
		B	NA	LCI	NR	NR	25.0	NR	65.0	NR	10.0
St-Onge et al., 2016 [37]	A	B	NA	HCI	NR	NR	53.5	NR	31.0	NR	17.0
		B	NA	LCI	NR	NR	54.6	NR	32.7	NR	14.0
Vlahoyiannis et al., 2018 [3]	B	A	B	HGI	801.2	178.0	88.9	2.4	2.7	16.9	8.4
		A	B	LGI	801.2	178.0	88.9	2.4	2.7	16.9	8.4
Daniel et al., 2019 [35]	B	A	A	HGI	1058	169.5	64.1	27.9	10.5	29.9	25.4
		A	A	LGI	1083	160.3	59.2	33.1	12.2	34.4	28.6

CHO = Carbohydrates; HCI = High Carbohydrate Intake; LCI = Low Carbohydrate Intake; ZCI = Zero Carbohydrate Intake; HGI = High Glycemic Index; LGI = Low Glycemic Index; NR = Not Reported; NA = Not Applicable.

**Table 3:** Sets of sleep-related derived variables.

Author, Year, Reference	Sleep Monitoring Method	Familiarization	Nights Recorded (Per Trial)	TST (min)	SE (%)	SOL (min)	WASO (min)	ROL (min)	N1 (min)	N1 (%)	N2 (min)	N2 (%)	N3 (min)	N3 (%)	REM (min)	REM (%)
Phillips et al., 1975 [24]	EEG	Y	2	Χ		Χ		Χ	Χ	Χ	Χ	Χ	Χ	Χ	Χ	Χ
Porter and Horne, 1981 [33]	PSG	Y	3			Χ		Χ	Χ		Χ		Χ		Χ	
Kwan et al., 1986 [36]	EEG	Y	2	Χ		Χ		Χ	Χ	Χ	Χ	Χ	Χ	Χ	Χ	Χ
Afaghi et al., 2007 [23]	PSG	Y	1	Χ	Χ	Χ	Χ	Χ		Χ		Χ		Χ		Χ
Afaghi et al., 2008 [2]	PSG	Y	1	Χ	Χ	Χ	Χ	Χ	Χ	Χ	Χ	Χ	Χ	Χ	Χ	Χ
Jalilolghadr et al., 2011 [34]	PSG	Y	1	Χ	Χ	Χ	Χ	Χ	Χ		Χ		Χ		Χ	
Lindseth et al., 2013 [31]	PSG	Y	1	Χ		Χ			Χ		Χ		Χ		Χ	
Lindseth and Murray, 2016 [32]	Actigraphy	N	4		Χ	Χ										
St-Onge et al., 2016 [37]	Actigraphy	Y	4	Χ	Χ	Χ	Χ									
Vlahoyiannis et al., 2018 [3]	PSG	Y	1	Χ	Χ	Χ	Χ	Χ	Χ	Χ	Χ	Χ	Χ	Χ	Χ	Χ
Daniel et al., 2019 [35]	Actigraphy	N	1	Χ	Χ	Χ	Χ									

EEG: Electroencephalography; PSG: Polysomnography; Y: Yes; N: No; TST: Total Sleep Time; SE: Sleep Efficiency; SOL: Sleep Onset Latency; WASO: Wake After Sleep Onset; ROL: REM Onset Latency; REM: Rapid Eye Movement.

**Table 4:** Metanalytic calculations for sleep-related variables according to CHO quantity and quality.

	CHO Quantity						CHO Quality					
	Hedges’ g	SE	z-Value	*p*	I^2^ Index	Results ^a^	Hedges’ g	SE	z-Value	*p*	I^2^ Index	Results ^a^
TST	0.01	0.09	0.08	0.936	12.33	NS	−0.25	0.16	−1.57	0.059	24.19	NS
SE	0.12	0.07	1.69	0.092	0	NS	−0.27	0.21	−1.27	0.203	63.09	NS
SOL	−0.13	0.11	−1.24	0.213	58.38	NS	0.58	0.47	1.24	0.213	83.54	NS
ROL	0.21	0.14	1.55	0.121	20.29	NS	0.07	0.20	0.34	0.731	46.08	NS
WASO	−0.22	0.12	−1.78	0.075	31.25	NS	0.11	0.20	0.57	0.569	58.09	NS
N1 min	0.06	0.1	0.60	0.551	0	NS						
N2 min	−0.02	0.09	−0.19	0.849	0	NS						
N3 min	0.37	0.07	5.13	<0.001	0	+						
REM min	−0.38	0.05	−8.05	<0.001	0	−						
% N1	0.02	0.12	0.16	0.872	0	NS	−0.21	0.16	−1.37	0.171	0.00	NS
% N2	0.03	0.08	0.39	0.698	0	NS	0.07	0.11	0.63	0.532	0.00	NS
% N3	0.51	0.06	8.90	<0.001	0	+	0.02	0.06	0.29	0.772	0.00	NS

^a^ plus sign indicates that the specific sleep measure was significantly more in the LCHO/LGI trial than HCHO/HGI at *p* < 0.05; N.S indicates no significant group differences. CHO: Carbohydrates; GI: Glycemic Index; TST: Total Sleep Time; SE: Sleep Efficiency; SOL: Sleep Onset Latency; WASO: Wake After Sleep Onset; ROL: REM Onset Latency; REM: Rapid Eye Movement; a: minus sign indicates that the specific sleep measure was significantly more in the HCHO/HGI trial than LCHO/LGI at *p* < 0.05.

## Data Availability

Data sharing not applicable.

## Supplementary Materials

The following are available online at https://www.mdpi.com/article/10.3390/nu13041283/s1, Figure S1: Funnel plots for Sleep Quantity, Figure S2: Funnel plots for Sleep Quantity, Table S1: Quality Assessment of the included studies using “QualSyst”.
