# Supplementary material for: A Systematic Review, Meta-Analysis and Meta-Regression on the Effects of Carbohydrates on Sleep
Source: Nutrients. 2021 Apr 14;13(4):1283. doi: 10.3390/nu13041283 (PMC8069918; doi:10.3390/nu13041283)
Supplement: Supplementary file 1 [file nutrients-13-01283-s001.zip › supplementary/Supplemental Figure S1.docx]

**Supplemental Figure S1.** Funnel plots for Sleep Quantity. A= Total Sleep Time; B=Sleep Onset Latency; C=REM Onset Latency ; D=N1 (min) ; E=N2 (min) ; F=N3 (min) ; G=REM (min) ; H=N1 (%) ; I=N2 (%) ; J=N3 (%); K=REM (%); L= REM attainment (%) ; M= REM attainment (min) ; N= REM attainment (%); O=REM attainment (min)
