# Supplementary material for: A Systematic Review, Meta-Analysis and Meta-Regression on the Effects of Carbohydrates on Sleep
Source: Nutrients. 2021 Apr 14;13(4):1283. doi: 10.3390/nu13041283 (PMC8069918; doi:10.3390/nu13041283)
Supplement: Supplementary file 1 [file nutrients-13-01283-s001.zip › supplementary/Supplemental Figure S2.docx]

**Supplemental Figure S2.** Funnel plots for Sleep Quantity. A= Total Sleep Time; B=Sleep Efficiency; C=Sleep Onset Latency; D=Wake After Sleep Onset; E=REM Onset Latency; F=N1 (%) ; G=N2 (%) ; H=N3 (%); I=REM (%); J= REM attainment (%) ; K= REM attainment (min) ; L= REM attainment (%); M=REM attainment (min)
