# Supplementary material for: A Systematic Review, Meta-Analysis and Meta-Regression on the Effects of Carbohydrates on Sleep
Source: Nutrients. 2021 Apr 14;13(4):1283. doi: 10.3390/nu13041283 (PMC8069918; doi:10.3390/nu13041283)
Supplement: Supplementary file 1 [file nutrients-13-01283-s001.zip › supplementary/Supplemental Table S1.docx]

**Supplemental Table S1.** Quality Assessment of the included studies using “QualSyst”.

| **Author** | **Date** | **Question described** | **Appropriate study design** | **Appropriate subject selection** | **Characteristics described** | **Random allocation** | **Researchers blinded** | **Subects blinded** | **Outcome measures well defined and robust to bias** | **Sample size appropriate** | **Analytical methods well described** | **Estimate of variance reported** | **Controlled for confounding** | **Results reported in detail** | **Conclusion supported by results** | **Rating (%)** | **Study Quality** |
| --- | --- | --- | --- | --- | --- | --- | --- | --- | --- | --- | --- | --- | --- | --- | --- | --- | --- |
| Phillips F | 1975 | 2 | 2 | 1 | 1 | 2 | 2 | 0 | 2 | 1 | 1 | 1 | 1 | 2 | 2 | 71.4 | Moderate |
| Porter JM | 1981 | 2 | 2 | 1 | 1 | 2 | 2 | 2 | 1 | 1 | 2 | 2 | 1 | 2 | 2 | 82.1 | Strong |
| Kwan RM | 1986 | 2 | 1 | 1 | 1 | 0 | 0 | 0 | 1 | 1 | 2 | 2 | 1 | 2 | 2 | 57.1 | Moderate |
| Afaghi A. | 2007 | 2 | 2 | 1 | 1 | 2 | 1 | 1 | 2 | 1 | 2 | 2 | 1 | 2 | 2 | 78.6 | Strong |
| Afaghi A | 2008 | 2 | 2 | 1 | 1 | 0 | 1 | 0 | 2 | 1 | 2 | 2 | 1 | 2 | 2 | 67.9 | Moderate |
| Lindseth G. | 2011 | 2 | 2 | 2 | 2 | 2 | 2 | 2 | 2 | 1 | 2 | 2 | 1 | 2 | 2 | 92.9 | Strong |
| Jalilolghadr S | 2011 | 1 | 2 | 2 | 2 | 2 | 1 | 0 | 2 | 1 | 2 | 2 | 1 | 2 | 2 | 78.6 | Strong |
| Lindsenth G. | 2016 | 2 | 2 | 2 | 2 | 2 | 2 | 2 | 2 | 1 | 2 | 2 | 1 | 2 | 2 | 92.9 | Strong |
| St-Onge MP | 2016 | 1 | 1 | 1 | 1 | 0 | 0 | 0 | 2 | 1 | 2 | 2 | 1 | 2 | 2 | 57.1 | Moderate |
| Vlahoyiannis A | 2018 | 2 | 1 | 1 | 1 | 2 | 2 | 2 | 2 | 1 | 2 | 2 | 1 | 2 | 2 | 82.1 | Strong |
| Daniel NVS | 2019 | 2 | 1 | 2 | 2 | 2 | 0 | 0 | 1 | 1 | 2 | 2 | 1 | 2 | 2 | 71.4 | Moderate |
